# Cardiovascular aspects of Noonan syndrome and related disorders

**DOI:** 10.1515/medgen-2025-2010

**Published:** 2025-04-08

**Authors:** Martin Zenker, Cordula M. Wolf

**Affiliations:** University Hospital Magdeburg Institute of Human Genetics Leipziger Str. 44 39120 Magdeburg Germany; German Centre for Cardiovascular Research Lazarettstr. 36 80636 München Germany

## Abstract

Noonan syndrome and other RASopathies constitute an important group of disorders to be considered in the differential diagnosis in individuals with congenital heart defects and hypertrophic cardiomyopathy. The cardiovascular phenotype of RASopathies is complex and comprises a spectrum of abnormalities, including not only congenital defects but also abnormalities affecting the lymphovascular system and other anomalies of the vascular system, which may emerge over the course of an individual’s lifetime. Affected individuals typically present with a syndromic phenotype, exhibiting additional physical symptoms outside of the cardiovascular system and neuropsychological deficits. Genetic testing of the established disease genes for RASopathies is an effective method for identifying the underlying genetic variant in the majority of cases. This approach is strongly recommended to facilitate a more precise prognosis and the potential for personalized targeted therapies. Screening for RASopathy-associated gene variants in individuals with isolated CHDs, HCM, or other isolated cardiovascular features outside the NS spectrum appears to have limited clinical utility. However, it should be noted that the RASopathy phenotype may be challenging to discern in cases of mild or oligosymptomatic involvement, or it may be obscured by the presence of severe medical conditions, particularly in very young children.

## Introduction

When Dr. Jacqueline Noonan first reported a series of patients exhibiting a distinctive pattern of clinical characteristics as having a “previously unrecognized syndrome of valvular pulmonary stenosis and multiple extra cardiac anomalies”, she recognized the pivotal role of cardiac involvement in this new syndrome [1]. Noonan syndrome (NS; MIM #PS163950) is the second most prevalent genetic syndrome associated with cardiac abnormalities after Down syndrome [2]. Cardiovascular involvement is present in 60–90 % of patients [3–6]. The prevalence of cardiac abnormalities varies between studies, most likely due to recruitment bias.

Cardiac abnormalities associated with NS can be classified into two main categories: Congenital heart defects (CHDs) represent errors of organogenesis and are typically present at birth. Hypertrophic cardiomyopathy (HCM), on the other hand, can manifest at various ages and may be understood as a maladaptive regulation of cardiomyocyte growth and differentiation. This review will also address two other aspects of cardiovascular health: functional cardiac abnormalities and vascular anomalies.

**Figure 1:**
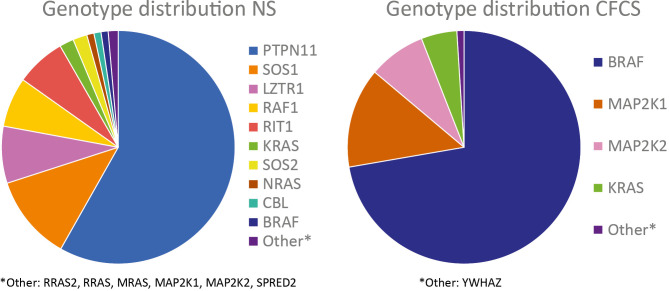
**Heterogeneity of NS and CFCS**. The diagrams show the contribution of the different known genes for NS and CFCS based on data from the NSEuroNet database and several larger published cohorts as specified in Table 1.

NS is regarded as the prototypical disease among the RASopathies. The term “RASopathies” was first introduced in 2009 [7] to describe a group of clinically overlapping multisystem disorders that share a common underlying molecular pathogenesis of constitutive dysregulation (i.e., for most causative variants, overactivation) of the RAS-MAPK signaling cascade due to pathogenic variants in genes encoding its components or modulators. RASopathies include NS and the less common clinically related disorders Costello syndrome (CS; MIM #218040), cardiofaciocutaneous syndrome (CFCS: MIM #PS115150), Noonan syndrome with multiple lentigines (NSML; MIM #151100), and Noonan syndrome-like disorder with loose anagen hair (NSLH; MIM #PS607721), also known as Mazzanti syndrome. Genes that are associated with the different RASopathy syndromes and their respective contribution to the total number of cases with a molecular diagnosis are given in **Table 1** and **Figure 1**. In accordance with their shared underlying pathogenesis, the spectrum of cardiac involvement is generally comparable across these entities [8]. It can be concluded that the “NS-like” spectrum of cardiac abnormalities represents the common phenotypic signature of dysregulated RAS signaling within the structures of the cardiovascular system. However, there are significant genotype-related differences in the prevalence and distribution of cardiac abnormalities, which will be discussed in more detail below. Other conditions, such as neurofibromatosis type 1 (MIM #162200), Legius syndrome (MIM #611431), and *RASA1*-related capillary malformation-arteriovenous malformation syndrome (MIM #608354) are also counted among the RASopathies. These conditions share the pathogenetic link with the RAS signaling pathway, but rarely exhibit cardiac involvement, possibly due to a less important role of their underlying genes in the developing heart. For this reason, they are not discussed in detail in this review.

## Congenital heart defects (CHDs)

As disorders of embryofetal cardiac development, CHDs are typically diagnosed shortly after birth, and in some cases even prenatally. The most prevalent CHDs in NS are pulmonary valve stenosis (PVS) and septal defects (**Table 1**). Calcagni et al. reported cardiac phenotypes in 440 molecularly characterized RASopathy patients (including NS, NSML, CFCS, and CS). Of these, 83 (19 %) did not present any cardiac defect [9]. A “common” CHD (PVS, septal defects) or hypertrophic cardiomyopathy (HCM) were observed in 312 subjects (71 %), while 45 (10 %) had “atypical” CHDs, most commonly mitral valve abnormalities in 31 subjects (7 %), followed by aortic valve anomalies (insufficiency or stenosis) in 16 (4 %), isolated anomalies of pulmonary arteries (stenosis, dilatation) in 7 (2 %), dilatation of coronary arteries in 7 (2 %), and aortic arch anomalies (coarctation, kinking, aortic root dilatation) in 5 (1 %). Tetralogy of Fallot and hypoplastic left heart syndrome have been reported anecdotally in NS [5,10].

**PVS** is the most prevalent CHD reported in over half of individuals affected by NS. PVS is also a typical finding in NS-related disorders but it occurs less frequently, with the frequency varying depending on the specific RASopathy type (**Table 1**). The prevalence of PVS in CFCS, as reported in different cohorts, ranges from 31 to 44 % [6,11]. In CS, PVS is present in 10–20 % of cases, it rarely has a severe grade and is more frequently associated with subvalvular and supravalvular pulmonary stenosis [6,10]. The prevalence of PVS in NSML is approximately 25 % [12]. PVS has a relatively high specificity for RASopathies, particularly for NS. Published retrospective and registry studies indicate that RASopathies account for 9–14 % of all cases with PVS [13,14]. The severity of PVS in NS was reported to be mild in approximately 60 % of cases, moderate in approximately 10 %, and severe in approximately 30 % [5]. A characteristic morphology of RASopathy-related PVS is well established among pediatric cardiologists, with the pulmonary valve leaflets frequently exhibiting dysplasia, thickening, and commissural fusion [5]. Besides PVS, both supravalvular and subvalvular (infundibular) pulmonary stenosis have also been documented in patients with NS and related disorders, though they are less prevalent. In a study conducted by Bell et al., 19 % of individuals with NS exhibited supravalvular pulmonary stenosis in conjunction with PVS or as an isolated anomaly [13].

**Septal defects** are the second most common CHDs in RASopathies affecting roughly one third of patients (**Table 1**). They are also common CHDs in various other syndromic conditions and as isolated cardiac anomalies in otherwise healthy individuals, and are therefore less specific for RASopathies. Among the septal defects, atrial septal defect (ASD) is the most common one, followed by ventricular septal defect (VSD), and atrioventricular canal defect (AVCD) [3,15]. AVCDs occur predominantly in the form of partial AVCDs, while complete AVCDs are exceedingly rare [5].

**Table 1:** Genotype distribution among RASopathies and cardiovascular phenotype according to genotype

**Disease**	**Gene**	**% for disease**	**PVS**	**ASD/VSD**	**HCM**	**Arrhythmia**	**Lymphatic**	**Reference**
**NS**	**All genes**	**100%**	**56%**	**27%**	**19%**	**4%**	**12%**	
	PTPN11	59%	**61%**	26%	6%	3%	9%	NSEuroNet, PMID: 11992261, 12634870
	SOS1	12%	**73%**	24%	14%	3%	12%	NSEuroNet, PMID: 17143282, 17143285, 18651097
	LZTR1 (AD)	7%	34%	22%	22%	3%	10%	NSEuroNet, PMID: 30368668, 30859559
	LZTR1 (AR)	1%	22%	35%	**75%**	0%	19%	NSEuroNet, PMID: 29469822, 30368668, 30859559
	RAF1	7%	13%	38%	**82%**	7%	11%	NSEuroNet, PMID: 17603482, 17603483
	RIT1	6%	**79%**	46%	**54%**	8%	**37%**	NSEuroNet, PMID: 26714497, 27101134
	KRAS	2%	52%	24%	34%	11%	7%	NSEuroNet, PMID: 17056636, 17366577
	SOS2	2%	18%	36%	18%	3%	**58%**	NSEuroNet, PMID: 32788663
	NRAS	1%	21%	9%	35%	3%	9%	NSEuroNet, PMID: 19966803
	CBL	1%	15%	5%	5%	3%	0% / NM	NSEuroNet, PMID: 25952305
	BRAF	1%	see below: CFC					
	RRAS2	<1%	*(8%)*	*(33%)*	*(0%)*	*(0%)*	*(0% / NM)*	NSEuroNet, PMID: 31130282, 31130285
	MAP2K1	<1%	see below: CFC					
	MAP2K2	<0,3%	see below: CFC					
	MRAS	<0,3%	*(17%)*	*(50%)*	*(****100%****)*	*(0%)*	*(0% / NM)*	PMID: 28289718, 31108500, 31173466, 34080768
	RRAS	<0,3%	Insufficient number of reported cases for statistical analysis					
	SPRED2	<0,3%	Insufficient number of reported cases for statistical analysis					
**NSML**	PTPN11*	100%**	27%	9%	**56%**	5%	0% / NM	NSEuroNet, PMID: 12058348, 15520399, 16523510, 17697839, 24775816
**NSLH**	**All genes**	**100%**	**33%**	**34%**	**25%**	**2%**	**10%**	
	SHOC2	93%	32%	34%	27%	2%	10%	NSEuroNet, PMID: 25331583
	PPP1CB	7%	40%	34%	3%	0%	7%	NSEuroNet
**CFC**	**All genes**	**100%**	**44%**	**21%**	**29%**	**3%**	**3%**	
	BRAF	73%	45%	25%	29%	4%	2%	NSEuroNet, PMID: 16439621, 16474404, 17704260, 17366577, 17551924, 18039946, 19206169
	MAP2K1	14%	42%	13%	21%	0%	0% / NM	NSEuroNet, PMID: 16439621, 17551924, 18039946, 19156172
	MAP2K2	8%	*(30%)*	*(0%)*	*(40%)*	*(0%)*	*(10%)*	NSEuroNet, PMID: 16439621, 18039946, 19156172
	KRAS	5%	see above: NS					
**CS**	HRAS	100%**	10%	7%	**60%**	**55%**	NM	GeneReviews

**Legend:** The percentage of cases ascribed to a particular gene was calculated from the NSEuroNet database (www.nseuronet.com). For more recently identified genes, a correction factor was used. The respective spectrum of cardiovascular manifestations was calculated using additional published data (PMIDs indicated in the column “Reference”). Numbers in italics and in parentheses indicate limited validity due to small cohort size (n<10). Significant genotype associations with cardiovascular phenotypes are highlighted by bold numbers. Percentage figures for arrhythmia and postnatal lymphatic anomalies may be less reliable because these features are recorded less stringently.*Specific PTPN11 variants are associated with NSML; ** Variations in this gene are considered to be the sole cause of the respective disease. ASD, atrial septal defect; Lymphatic, postnatal lymphatic anomalies; NM, not mentioned; PVS, pulmonary valve stenosis; VSD, ventricular septal defect.

**Other CHDs:** The full range of RASopathy-related CHDs encompasses a multitude of other anomalies, which may manifest concurrently with one of the aforementioned defects or in isolation [9]. The mitral valve is the second most commonly affected valve with congenital anomalies (predominantly mitral valve dysplasia and mitral valve insufficiency) [9]. The co-occurrence of congenital dysplasia of two or more cardiac valves, referred to as congenital polyvalvular disease, has been documented in a minority of patients [4,16]. Congenital left-sided obstructive cardiac abnormalities (excluding those caused by HCM) have been observed in up to 12 % of patients with NS. They include aortic stenosis at the valvular or subvalvular level, as well as coarctation of the aorta (CoA) [6].

For individuals with an isolated CHD lacking other extracardiac anomalies suggestive of NS, current experience suggests that the probability of a RASopathy being the underlying cause is very low [14, 17]. The majority of cases in which a RASopathy is diagnosed through genetic testing for a seemingly isolated heart defect are not truly nonsyndromic occurrences, but rather result from a lack of recognition.

## Hypertrophic cardiomyopathy (HCM)

Hypertrophic cardiomyopathy (HCM) denotes abnormal thickening of the myocardium which is not secondary to increased cardiac workload. HCM can result in ventricular outflow tract obstruction, diastolic dysfunction due to impaired filling of the ventricular cavity during diastole, and an increased risk of arrhythmias and sudden cardiac death. HCM has a reported prevalence of 10–29 % in NS [5] (**Table 1**). Differences in prevalence between reported cohorts of patients may be due to inconsistencies in the criteria and definitions used for the diagnosis of HCM. The frequency of HCM is strongly correlated with the NS genotype, as will be discussed in further detail below. Among NS-related disorders, HCM is particularly prevalent in NSML and CS, affecting more than half of patients (**Table 1**). The prevalence of HCM in CFCS is comparable to or slightly higher than that in NS (30–40 %), yet it rarely manifests in a severe form [6,11].

RASopathy-associated HCM differs from other genetic (e.g., sarcomeric) cardiomyopathies in terms of its age of onset and clinical course. A number of studies have demonstrated that the onset of HCM in individuals with RASopathy-associated HCM typically occurs at an early age, with a significantly younger age of manifestation than that observed in sarcomeric HCM [3,18–23]. Signs of HCM may already be present prenatally. In comparison to HCM of other etiologies, HCM in RASopathies has also been documented to be distinguished by a more pronounced degree of left ventricular hypertrophy and a higher prevalence and severity of left ventricular outflow tract obstruction [6]. Biventricular hypertrophy is relatively common and may manifest in the presence or absence of PVS. It has been proposed that the presence of additional cardiac findings such as coexisting CHD, concomitant right ventricular hypertrophy or outflow tract obstruction, and extreme QRS axis deviation should trigger consideration of a RASopathy as the potential cause of HCM in children [19].

On long-term follow-up, clinical functional status can improve and left ventricular posterior wall thickness indexed to body surface area can decrease in NS-associated HCM, in contrast to sarcomeric HCM [20]. Similar observations have been made for NSML-associated HCM: In a cohort of 42 patients, Monda et al. reported absolute and relative regression (i.e. stability of left ventricular wall thickness with somatic body growth) of NSML-associated HCM during a median follow-up of 3.7 years in 31 % and 21 % of cases, respectively, whereas progression was only seen in 21 % [24]. Nonetheless, RASopathy-associated HCM represents a potentially life-threatening condition, particularly when accompanied by congestive heart failure during infancy. Reported outcomes for young infants with NS-related HCM and early-onset heart failure were unfavorable. Wilkinson et al. reported a mortality of 22 % at 1 year for NS-associated HCM, and when congestive heart failure was present in the first 6 months of life, mortality was as high as 69 % in the first year after HCM diagnosis [23]. Subjects with RASopathy-associated HCM but without symptoms of heart failure are at risk of sudden cardiac death, which has been observed more frequently among adolescents and young adults, while nonarrhythmic deaths occurred primarily in infancy [22,25,26]. The estimated cumulative 10 year incidence of sudden cardiac death or equivalent events in patients with RASopathy-associated HCM was 5–9 % [22,26]. In general, morbidity and mortality from HCM in NSML appears to be quite similar to NS [24]. Boleti et al. observed that patients with NSML exhibited higher left ventricular outflow tract gradient values compared to those with other RASopathies. These values were identified as predictors of an unfavorable outcome together with the presence of congestive cardiac failure, occurrence of non-sustained ventricular tachycardia, and the maximal left ventricular wall thickness [19].

In many diagnostic laboratories, genes associated with RASopathies are incorporated into diagnostic cardiomyopathy multigene panels. The diagnostic yield of pathogenic variants in RASopathy genes in cardiomyopathy cohorts varies considerably between published studies and is dependent on several factors, including patient selection, age, type of cardiomyopathy, and other variables. In pediatric HCM cohorts, the prevalence of RASopathy-associated HCM has been reported to vary between 4 % and up to 42 % in cases presenting before the age of one year [24,27,28]. However, while a number of studies have reported causative variants in RASopathy genes in patient cohorts examined for apparently isolated HCM [27,29,30,31] their contribution in well-phenotyped, truly non-syndromic cases is probably quite low.

## Electrocardiographic abnormalities and arrhythmias

It is well established that **electrocardiography (ECG) abnormalities** are highly prevalent in individuals with NS. The most common findings are extreme axis deviation, left anterior hemiblock, and an RSR’ pattern in lead V1 [34]. Vos et al. reviewed on the first ECG available from 95 patients with NS the presence of specific electrocardiographic features: left axis deviation, large S-waves in the right precordial leads, small R-waves in the left precordial leads, abnormal Q-waves, and/or wide QRS complexes. One or more of these NS-related ECG features were observed in 63 % of patients. ECG abnormalities were seen in the majority (76 %) of patients with CHD or HCM, but were also increased above baseline in patients without abnormal structural cardiac findings (42 %) [35]. Similarly, ECG abnormalities are also frequently observed in NSML, predominantly in association with HCM. Furthermore, Limongelli et al. reported prolonged QTc intervals (23 %), repolarization abnormalities (42 %), and conduction defects (23 %) in a patient cohort with NSML [36]. Hauptmeijer et al. compared ECG abnormalities in RASopathy-associated HCM vs. sarcomeric HCM. They found a negative aVF (indicative for extreme axis deviation) in 83 % of patients with NS-associated HCM, which was notably higher than the 27 % observed in primary sarcomeric HCM. An extreme right axis deviation was observed exclusively in patients with NS-associated HCM. These findings indicate that specific ECG abnormalities are intrinsic to the NS heart and that the ECG can assist in differentiating between NS-related and sarcomeric HCM [37].

**Arrhythmias** may occur in RASopathies with, but sometimes also independently of, HCM. They include, in particular, multifocal or ectopic atrial tachycardia, which often has a perinatal presentation, as well as other less specific types of arrhythmias [20,38]. Atrial tachyarrhythmias are particularly prevalent in patients with CS, occurring in over 50 % of cases. These arrhythmias typically have a benign course and often resolve within the first year of life, either with or without medical therapy. Non-reentrant supraventricular tachycardias and tachyarrhythmias have rarely been observed in NS and NSML, with reports of such occurrences in patients with pathogenic variants in *RAF1*, *PTPN11*, and *SOS1* [38]. While the majority of cases harbored genetic variants known to be highly associated with HCM, it has been noted that non-reentrant atrial tachycardias may also occur independently of HCM [38]. Ventricular arrhythmias are very rare in the absence of underlying structural cardiac abnormalities and they have predominantly been observed in individuals with HCM. Non-sustained ventricular tachycardia and unexplained syncope have been identified as predictors for sudden cardiac death or equivalent events in RASopathy-associated HCM [19]. While earlier studies indicated that the risk of sudden cardiac death or severe arrhythmic events was lower in NS/RASopathies than in sarcomeric HCM [15,20], more recent studies suggested that this might have been an underestimation and observed a quite similar prevalence of sudden cardiac death or equivalent events of nearly 5 % [19,22]. In this context, it is noteworthy that studies have reported a lower incidence of implantable cardioverter-defibrillator (ICD) insertions in patients with RASopathy-associated HCM compared to sarcomeric HCM [20,22]. Together, these findings highlight the importance of considering ventricular arrhythmias and the risk of sudden death in individuals with RASopathy and tailoring diagnostic and therapeutic strategies accordingly [19].

## Vascular malformations and abnormalities

A variety of vascular anomalies have been linked to RASopathies. Most importantly, **lymphovascular malformations** have to be considered as typical and common vascular anomalies in this group of diseases. The nature of RASopathy-associated lymphovascular anomalies is consistent with that of a central conducting lymphatic anomaly (CCLA). Pieper et al. identified thoracic duct dysplasia, intercostal reflux, and pulmonary/pleural lymphatic perfusion as distinctive features in individuals with NS presenting with chylothorax and/or pulmonary lymphangiectasia [39]. Pleural effusions/chylothorax are characteristic manifestations of RASopathy-associated lymphovascular anomalies, which may manifest prenatally or at any point during the lifespan. Fetal nuchal edema may be regarded as the earliest indicator of a developmental anomaly of the lymphovascular system, and it may remain the only sign. The spectrum of lymphatic edema and effusions, however, is broad. Swarts et al. determined a lifetime prevalence of lymphatic anomalies of 37 % in NS [40].

Other vascular anomalies, such as arteriovenous malformations (AVMs) or cavernous hemangiomas, have been infrequently documented in patients with RASopathies [41]. The observation that somatic variants in RAS-MAPK pathway genes are frequent drivers of sporadic AVMs and other vascular anomalies [42] indicates that the association of such anomalies with germline variants is not merely coincidental.

**Arterial dilatation or aneurysms** of the coronary and carotid arteries, as well as of the aorta, have more recently been identified as being associated with RASopathies at a low frequency [5,6]. The prevalence and clinical significance of such findings remain poorly defined. A retrospective study of ascending aortic anatomy from routine echocardiograms performed on patients with NS demonstrated that aneurysms (defined as Z score ≥2) were present in approximately 20 % of patients with NS, often presenting during childhood and progressing over time [43]. Aortic dissection, however, has very rarely been reported in individuals with NS. Arterial dilation or aneurysm has in particular been observed at the site of the coronary arteries in patients with different types of RASopathies [5,6,9,36,44]. In a large patient cohort of patients with RASopathies, Calcagni et al. identified 7 cases of coronary artery aneurysms among 440 individuals (1.6 %) [9]. In a smaller cohort of patients with NSML, similar abnormalities were observed in 4 of 26 individuals (15 %) [36]. In the majority of reported cases, coronary artery ectasia was observed as a concomitant abnormality in patients with HCM. However, the occurrence of coronary artery ectasia in patients without HCM or other coexistent cardiovascular abnormalities indicates that this anomaly may also be related to the dysregulation of the RAS-MAPK pathway itself [6].

**Cerebral arteriopathy and Moyamoya disease** have been reported in a limited number of individuals with various types of other RASopathies including neurofibromatosis type 1. Cases of Moyamoya disease have been observed in association with NS, followed by NSLH, CFCS, and CS [41]. A particularly elevated risk of cerebrovascular complications and Moyamoya disease appears to be associated with pathogenic variants of *CBL*. Patients may present with apparently isolated cerebrovascular disease and only subtle signs of a RASopathy [45,46]. The pathogenesis of arterial occlusive disease in RASopathies is thought to involve aberrant proliferation of cells within the arterial wall [5]. In patients with pathogenic *CBL* variants, however, vasculitis appears to be the primary mechanism driving the vasculopathy. Bone marrow transplantation has been shown to halt the progression of vascular disease in these patients [47].

## Prenatal cardiovascular manifestations

Cardiovascular abnormalities in RASopathies may be present from the earliest stages of fetal development and may be the indication for prenatal genetic testing. Fetal abnormalities related to lymphovascular defects are the most frequent prenatal manifestations of all RASopathies. They can range in severity from simple nuchal edema to hydrops fetalis. In cases of fetal nuchal edema/increased nuchal translucency with a normal karyotype, the probability of identifying a RASopathy as the underlying cause is between 2 and 5 %. In cases of more severe manifestations, such as cystic hygroma, fetal pleural effusions, or hydrops fetalis, the likelihood of a RASopathy diagnosis may increase to >20 % [48–51]. Large size and persistence of fetal nuchal edema are also associated with a higher likelihood of a RASopathy-causing variant [48,51]. Fetal cardiac anomalies (CHD or HCM) are less frequently the underlying cause of a prenatal RASopathy diagnosis.

## Genotype-phenotype correlations

NS and related disorders are genetically heterogeneous; the 20 known genes account for very different proportions of cases (**Table 1**, **Figure 1**). While the typical range of cardiac and vascular abnormalities associated with RASopathies may be observed across the spectrum of RASopathy types and genetic etiologies, there are notable genotype-phenotype correlations. They are in particular regarding the prevalence of HCM which is highest for *RAF1* (>80 %), followed by NSML-related *PTPN11* variants and *RIT1* (>50 %). Pathogenic variants in *MRAS* and biallelic variants in *LZTR1* (which cause the autosomal recessive form of *LZTR1*-related NS) are also strongly associated with HCM (**Table 1**). In contrast, *PTPN11*-related NS and *SOS1* variants have been demonstrated to be negatively associated with HCM [5,6]. Pathogenic variants in *RIT1* have been found to predispose to both HCM and PVS [52]. Not only the presence or absence of HCM varies depending on the underlying genetic cause but also its expression and course. Boleti et al. noted that patients with NSML and individuals with *RAF1* variants had more severe left ventricular hypertrophy and higher resting left ventricular outflow tract gradients, while patients with CS and CFCS had lower maximal left ventricular wall thicknesses and were less likely to have resting left ventricular outflow tract obstruction [19]. Moreover, even within the same disease category and gene, the expression of HCM may be correlated with individual variants. The association of *PTPN11* variants altering codon 510 with early-onset, severe left ventricular hypertrophy and adverse outcomes is one example [24]. In patients with *SHOC2*-related NSLH, semilunar valve abnormalities have recurrently been reported, while PVS is less common [53]. PVS is notably uncommon in patients with *RAF1*-related NS. Pathogenic variants of *RIT1* and *SOS2* have been linked to a higher prevalence of lymphovascular anomalies [39,52,54]. The prevalence of septal defects instead does not appear to vary significantly between different genetic etiologies of RASopathies (**Table 1**).

## Molecular pathogenesis of RASopathy-associated cardiovascular defects

Despite a plethora of animal and *in vitro* models that have been developed for studying the pathogenesis of RASopathy-associated abnormalities [55], the complex molecular pathogenesis of cardiovascular anomalies remains incompletely understood. With regard to the pathogenesis of RASopathy-related CHDs, studies on mouse models expressing NS-associated *Ptpn11* variants studies indicate that elevated RAS-MAPK activation driven by overactive Ptpn11 results in aberrant endocardial cushion formation, eventually leading to valvuloseptal defects that are typical of NS [56,57]. In the lymphatic system, dysregulation of the EPHB4-RASA1-mediated signaling via the RAS-MAPK pathway may contribute to abnormal lymphatic valve development and remodeling [58].

While overactivated signaling through MAPKs is generally thought to be the critical molecular driver across the group of RASopathies, it has become clear that interconnected alternative or noncanonical effector pathways also play a role in cardiac pathophysiology. Notably, evidence suggests that the co-regulation of the PI3K-AKT-mTOR pathway may be involved in the pathophysiology RASopathy-associated HCM [59]. While enhanced MEK-ERK activity has been demonstrated to be critical for causing HCM in *Raf1* and *Kras* mutant mice [60,61], studies on mouse models with the NSML-causing *Ptpn11* variants p.(Tyr279Cys) and p.(Gln510Glu) have indicated that NSML-associated HCM is mainly driven by activation of the PI3K-AKT-mTOR pathway [62,63]. Inhibition of mTOR has been observed to rescue the HCM phenotype in these models. Nevertheless, it is uncertain whether a rigid dichotomy between PI3K-AKT-mTOR-driven NSML-related and RAS-MAPK-driven NS-related HCM accurately reflects the biological reality. The molecular mechanisms downstream of these central signaling pathways are even less understood. Studies using various transgenic animals or *in vitro* models have produced diverse and sometimes inconsistent results. Investigations of cardiac tissue samples from patients, as well as myocardial cells or tissue constructs generated from patient-derived induced pluripotent stem cells (iPSCs), have indicated that abnormal cell cycle regulation and a lack of maturation may result in cardiomyocyte hyperplasia, which may be a significant contributing factor to the increased myocardial mass observed in these patients [59,64]. Other studies, however, have identified cardiomyocyte hypertrophy (enlargement) and myofibrillar disarray as characteristic features of iPSC-derived *RAF1*- or *LZTR1*-mutated cardiomyocytes and patient cardiac tissue samples [65–67]. A number of studies have documented abnormal calcium transients and calcium handling in cardiomyocytes harboring pathogenic variants in RASopathy genes [64–68]. However, it remains unclear whether the observed abnormalities are a direct consequence of dysregulated signaling pathways or secondary to abnormalities in other properties of mutated cells. A variety of mediators and effector pathways downstream of RAS have been proposed to play a critical role in different models of HCM, such as MEK5-ERK5 [59] RAC-STAT3, and cytokines [68,69]. In sum, the available evidence indicates that the mechanisms underlying myocardial hypertrophy are complex, and no unifying mechanistic model has yet been established. The observed heterogeneity in reported findings is likely attributable to both the genetic heterogeneity of RASopathies and the wide spectrum of model systems studied and methods employed. This underscores the need of further research.

## Implications for patient management

The growing body of knowledge regarding the RASopathy-associated cardiovascular phenotype, genotype-phenotype correlations, and molecular pathomechanisms has significant implications for patient management, follow-up, and treatment. In general, every individual newly diagnosed with NS or a related disease should undergo a comprehensive cardiology evaluation [34]. Subsequent cardiac surveillance should be tailored to the individual cardiac phenotype and the specificities of the underlying genetic cause. In light of the potential for postnatal cardiac phenotypes to emerge, it is advisable to conduct at least an annual cardiac evaluation until the age of five, even in the absence of initial cardiac abnormalities. Subsequent cardiology visits should be conducted at regular five-year intervals throughout adulthood [34].

Concerning the management of CHDs, strategies generally follow established guidelines regardless of the underlying RASopathy [5]. Nevertheless, there is some evidence that RASopathy-related PVS differs in its nature and response to treatment from PVS of other etiologies. Due to valve dysplasia, percutaneous balloon valvuloplasty is less likely to result in a sufficient reduction of the pulmonary gradient. This, in turn, results in the need for reintervention in up to two-thirds of cases [5,15,70]. Surgical treatment of PVS appears to have a similar success rate in children with NS and without [6]. The potential for complex structural anomalies, including the co-occurrence of or susceptibility to the development of HCM with cardiac valve abnormalities, necessitates tailored treatment decisions and may also contribute to an increased risk for reintervention and peri-interventional complications [3,6].

An enhanced understanding of arrhythmic complications in NS necessitates a re-evaluation of risk assessment and decisions regarding preventive management strategies, such as ICD implantation. In patients with RASopathy-associated HCM, unexplained syncope and non-sustained ventricular tachycardia were identified as predictors of sudden cardiac death risk, while echocardiographic parameters demonstrated less predictive value [26]. In light of the findings of recent studies indicating that the risk of sudden cardiac death or equivalent events is comparable between patients with RASopathy-associated HCM and those with sarcomeric HCM, the lower incidence of ICD insertions in the former population gives rise to concerns about potential under-treatment [19,20,22].

During follow-up, it is important to be aware of the potential for the emergence of aneurysms of the coronary, carotid arteries, and aorta vascular over the course of a patient’s lifetime. However, the clinical significance and long-term consequences of such anomalies remain uncertain, and recommendations regarding specific measures and examination intervals for surveillance are controversial [5,6]. The use of antiplatelet or anticoagulant medications to prevent coronary artery thrombosis in patients with coronary artery dilation should be considered [6].

Two common comorbidities in RASopathies are of particular relevance for periprocedural care of patients, particularly in the context of surgical interventions. These include lymphovascular complications and bleeding diathesis [5]. Developmental lymphovascular defects can be unmasked by open heart surgery, resulting in postoperative chylothoraces or, more rarely, chylous pericardial effusions. The reported prevalence of postoperative chylothorax in patients with NS undergoing cardiac surgery has been reported to be approximately 10 % [71]. Additionally, postprocedural pericardial or pleural effusion has been identified as a significant cause of reintervention [3]. Furthermore, concomitant chylothorax or pulmonary lymphangiectasia may exacerbate cardiovascular compromise in infants with CHDs or HCM, and vice versa. A bleeding diathesis has been reported in approximately two-thirds of patients with NS and may be associated with various abnormalities in the hemostatic system, including thrombocytopenia, platelet dysfunction, von Willebrand disease, and/or coagulation factor deficiencies [72]. Although severe perioperative bleeding events are uncommon in individuals with NS and related disorders, they may occur in patients who did not have abnormalities in basic coagulation screening tests [73].

Concerns have been raised that growth hormone therapy, which is approved for the treatment of short stature in NS, may have adverse effects on HCM [74]. Clinical studies examining the cardiac outcomes in patients undergoing growth hormone treatment did not identify any treatment-related adverse effects [75,76]. However, the investigated cohorts were relatively small, which limits the ability to exclude the possibility of adverse effects on HCM. It is recommended that patients with pre-existing HCM who are undergoing growth hormone therapy receive closer cardiology monitoring.

The treatment of RASopathy-associated HCM is primarily symptomatic and includes the use of beta-blockers, which are typically considered the first-line medication, as well as non-vasodilating calcium antagonists or disopyramide [6]. Surgical myectomy and heart transplantation represent further steps in the escalation of treatment options; however, their applicability is limited in very young children. In recent years, new treatment options have emerged through the understanding of the underlying pathophysiology by directly targeting the RAS-MAPK or the PI3K-AKT-mTOR pathways [5,6,55,77]. MEK inhibition has been demonstrated to reverse or ameliorate multiple elements of the phenotypes observed in animal and *in vitro* models of RASopathies. In particular, treatment with MEK inhibitors was observed to reverse hypertrophic myocardial changes in mouse models for *Raf1*- and *Kras*-related NS [60,61]. Additionally, the same treatment was found to be effective in iPSC-derived cardiomyocytes or myocardial tissue constructs harboring the recurrent *RAF1* variant p.(Ser257Leu) [68,69]. In contrast, in a mouse model for NSML with the recurrent *Ptpn11* variant p.(Tyr279Cys), inhibition of the PI3K-AKT-mTOR axis, either with the mTOR inhibitor rapamycin or the AKT inhibitor ARQ 092, demonstrated beneficial effects on cardiac hypertrophy [62,78]. This was seen as proof of the principle that NSML-related HCM may be driven more by PI3K-AKT-mTOR hyperactivation, as discussed above. Another study employing the Bcl-Abl and Src kinase family inhibitor dasatinib documented the alleviation of HCM in this same model [79].

Off-label treatment with the MEK inhibitor trametinib was first reported to improve clinical status and to result in regression of left ventricular hypertrophy in two infants with *RIT1*-associated HCM [80]. A very recent retrospective analysis of 30 patients with RASopathy-associated HCM (the majority having pathogenic variants in *RAF1* and *RIT1*) demonstrated a significant improvement in survival without cardiac surgery for outflow tract resection or heart transplantation, as well as notable clinical (Ross classification), neurohumoral (N-terminal pro-hormone brain natriuretic peptide), and echocardiographic (outflow tract gradient, myocardial hypertrophy) improvement over time [81]. Observations in individuals and small case series suggest that MEK inhibition might also be useful for the treatment of lymphovascular abnormalities and arrhythmia occurring in NS [5,6]. The current body of published evidence for the beneficial effects of PI3K-AKT-mTOR axis inhibition in patients with NSML-related HCM remains limited. In a single case report, Hahn et al. described a patient with NSML who exhibited clinical improvement following treatment with the mTOR inhibitor everolimus [82]. Additional small molecule inhibitors acting at different levels of the signaling cascades are available, and some of them have been utilized in preclinical RASopathy models [5,6,55]. Strategies to inhibit overactivated signaling pathways in RASopathies may not be limited to the use of small molecule inhibitors. Alternative approaches such as antisense oligonucleotides directed against the mutated RNA or genome editing may emerge in the future. Hanses et al. described a specific genetic engineering approach in a preclinical model of NS-associated HCM caused by a homozygous intronic *LZTR1* variant, suggesting that future therapies may be highly personalized [67].
